# Alternative Splicing of Serum Response Factor Reveals Isoform-Specific Remodeling in Cardiac Diseases

**DOI:** 10.3390/genes16080947

**Published:** 2025-08-11

**Authors:** Sayed Aliul Hasan Abdi, Gohar Azhar, Xiaomin Zhang, Shakshi Sharma, Mohib Hafeez, Jeanne Y. Wei

**Affiliations:** Department of Geriatrics, Donald W. Reynolds Institute on Aging, University of Arkansas for Medical Sciences, Little Rock, AR 72205, USA; shasan@uams.edu (S.A.H.A.); azhargohar@uams.edu (G.A.); zhangxiaomin@uams.edu (X.Z.); ssharma@uams.edu (S.S.); mhafeez@uams.edu (M.H.)

**Keywords:** cardiovascular disease, alternative splicing, serum response factor, RNA-seq

## Abstract

**Background:** Alternative splicing is an important mechanism of transcriptomic and proteomic diversity and is progressively involved in cardiovascular disease (CVD) pathogenesis. Serum response factor (SRF), a critical transcription factor in cardiac development and function, may itself undergo splicing regulation, potentially altering its function in disease states. **Objective:** The objective of this study is to identify SRF-associated alternative splicing events in cardiac pathological conditions and examine regulatory interactions with splicing factors using RNA-seq data. **Methods:** Three human heart RNA-seq databases (PRJNA198165, PRJNA477855, PRJNA678360) were used, comprising various cardiac conditions like non-ischemic cardiomyopathy (NICM), ischemic cardiomyopathy (ICM), dilated cardiomyopathy (DCM), and heart failure with reduced ejection fraction (HFrEF), with and without left ventricular assist device (LVAD) support. Splicing events were identified using the rMATS tool, and correlation analyses were performed between SRF and predicted splicing factors. Functional enrichment of SRF-correlated genes was assessed via Gene Ontology (GO) and KEGG pathways. **Results:** The skipped exon (SE) events were the predominant splicing type across all datasets. SRF chr6, including (Exon 2, 43,173,847–43,174,113), (Exon 4, 43,176,548–43,176,667), and (Exon 5, 43,178,294-43,178,485), were most frequently involved in SE and mutually exclusive exon (MXE) events across multiple heart failure subtypes. Correlation analysis revealed strong positive associations between SRF and several splicing factors (HNRNPL, HNRNPD, SRSF5, and SRSF8). GO and KEGG analyses revealed enrichment of muscle development, sarcomere structure, lipid metabolism, and immune signaling pathways. **Conclusions:** Our study shows that SRF is subject to extensive alternative splicing in heart failure, particularly at Exon 2 and Exon 5, suggesting isoform-specific roles in cardiac remodeling. The strong co-expression with specific splicing factors delineates a regulatory axis that may explain the pathological transcriptome in cardiomyopathy. These findings provide a foundation for exploring splicing-based biomarkers and therapeutic targets in cardiac pathology for SRF.

## 1. Introduction

Cardiovascular diseases (CVDs) are a root cause of death and illness around the world, affecting millions of people every year. Even with decades of research, as well as impressive clinical advances, we still do not fully understand what takes place at the molecular level when it comes to cardiac disease [1]. The evidence suggests that cardiac pathological changes involve complex gene expression patterns, including post-transcriptional regulatory mechanisms, such as alternative splicing. In fact, alternative splicing might play a key role in shaping the cardiac transcriptome in both health and disease [2]. This process, which allows a single gene to generate multiple transcripts through alternative splicing, results in a huge amount of proteomic diversity and functional capacity, especially in cardiac tissues that require the precise regulation of structural and signaling proteins [3].

Alternative splicing is a biological mechanism by which different combinations of exons are fused together during the maturation of pre-mRNA, creating multiple mRNA isoforms from the same gene [4]. This whole process is not random; it is tightly regulated by several cis-regulatory regions and trans-acting factors, including RNA-binding proteins (RBPs) and specific splicing factors. When these controls are disrupted, the splicing process can fail, leading to misprocessed RNA, and this kind of splicing error has been linked to several diseases, including cancer, neurodegenerative disorders, and, importantly, various cardiac conditions [5,6,7]. Proper regulation of splicing is required in the heart to support normal growth, contractility, and adaptive responses to stress. Splicing dysregulation has been linked to a variety of cardiac diseases, including hypertrophic and dilated cardiomyopathy, arrhythmias, and heart failure [3,7,8].

One of the key regulators of gene expression within cardiac tissue is SRF, which is a transcription factor better known for its central role in the regulation of genes involved in the growth of cells, cytoskeletal organization, and muscle differentiation. SRF is specifically crucial in the heart because it directs the generation of various structural and contractile proteins, as well as signaling pathways that respond to mechanical and neurohumoral stimuli. Altered SRF expressions or activity have been reported for various cardiac conditions such as poor cardiac remodeling, cardiomyocyte hypertrophy, fibrosis, and impaired contractility. While SRF has generally been recognized for its transcriptional regulatory roles, there is a rising interest in understanding its interactions with splicing regulators and its possible role in modulating or being influenced by alternative splicing pathways [9,10].

The human heart is a highly dynamic organ with specific chamber functions that respond to physiological and pathological stimuli. This functional and spatial diversity necessitates highly controlled and context-specific gene expression systems. According to recent research, alternative splicing patterns were found to be vastly different in various heart chambers (e.g., the atria vs. ventricles), types of cardiomyopathies (e.g., ischemic vs. non-ischemic types), and stages of heart failure. Furthermore, mechanical therapies such as left ventricular assist devices (LVADs), which are used to help patients with end-stage heart failure, have been demonstrated to affect molecular signaling and may also modify the cardiac splicing landscape [11,12].

Advances in high-throughput sequencing technology, and more precisely RNA sequencing (RNA-seq), have transformed transcriptome profiling, allowing for the in-depth analysis of alternative splicing events across tissues and condition-specific contexts. Although there is a growing awareness of alternative splicing as an important component of gene regulation in the heart, there is limited information on how splicing events are controlled in disease contexts or which splicing factors are involved. Specifically, the relationship between SRF and the splicing event in human heart failure has not been adequately studied. Addressing this gap demands not only identifying differently spliced events, but also mapping their regulatory landscape, which includes identifying critical splicing factors that interact with SRF or regulate SRF [12,13,14].

In this context, our research is motivated by a desire to better understand how alternative splicing, specifically events linked with SRF, contributes to the molecular pathophysiology of cardiovascular diseases. We intend to extensively investigate the landscape of SRF-related splicing events in various cardiac illness scenarios and identify the splicing factor networks that may govern these alterations. We hope to provide a complete perspective of alternative splicing dynamics in the human heart by exploiting publicly accessible RNA-seq datasets covering various cardiac diseases, such as ischemic and non-ischemic cardiomyopathy, LVAD-supported hearts, and chamber-specific tissue samples. Furthermore, by combining statistical and computational analysis with network biology methodologies, the aim of this study is to discover potential splicing regulators that may be driving transcriptomic alterations observed [14,15].

Finally, this study enhances our understanding of the complex regulatory systems that underpin cardiovascular disease, emphasizing the significance of alternative splicing as both a diagnostic and a possible modulator of cardiac function. It lays the framework for future experimental validation and therapeutic investigation, with the modification of splicing patterns potentially opening up new paths for the treatment of heart failure and other kinds of cardiomyopathy. In this study, we performed a high-computational analysis of publicly available human heart RNA-seq datasets covering diverse cardiac conditions, such as ischemic and non-ischemic cardiomyopathy, dilated cardiomyopathy, and heart failure with a reduced ejection fraction, both with and without LVAD support.

## 2. Methods

### 2.1. Data Source and Processing

Three raw RNA-Seq datasets, PRJNA678360, PRJNA198165, and PRJNA477855, were downloaded from the Sequence Read Archive (SRA) database https://www.ncbi.nlm.nih.gov/sra, (accessed on 7 June 2025). The details of the data are presented in Table 1. Data processing was performed on an Ubuntu 22.04.3 (64-bit) system. Sequence quality was assessed using FastQC (v0.11.9), and adapter trimming was conducted using trimmomatic (v0.39). Cleaned reads were aligned to the reference genome using the STAR aligner (v2.7.10a), which generates sorted BAM files appropriate for downstream analysis. Subsequently, statistical analyses and data visualization were performed using R (version 3.5.1), https://www.r-project.org/, (accessed on 7 June 2025) [16,17,18].

To investigate the alternative splicing patterns of serum response factor (SRF), the replicate Multivariate Analysis of Transcript Splicing (rMATS, v4.1.2) tool was used. This robust method allows for the detection and quantification of splicing events from replicate RNA-Seq data by comparing different biological conditions. Input files included the aligned BAM files and the corresponding GTF annotation file for the reference genome (GRCh38/hg38) [19].

To study the alternative splicing patterns of serum response factor (SRF), the replicate Multivariate Analysis of Transcript Splicing (rMATS) tool was used. This robust technique enables the detection and quantification of splicing events in duplicate RNA-Seq data by comparing various biological circumstances. The aligned BAM files and the reference genome’s GTF annotation file were used as input files [19].

rMATS was used to identify five distinct types of alternative splicing events:

Skipped Exon (SE): An exon is included in one transcript isoform but skipped in another.

Alternative 5′ Splice Site (A5SS): An alternative donor site is used at the 5′ end of the intron, resulting in a shorter or longer upstream exon.

Alternative 3′ Splice Site (A3SS): An alternative acceptor site is used at the 3′ end of the intron, leading to variation in the downstream exon length.

Mutually Exclusive Exons (MXE): Two exons are spliced in a mutually exclusive manner but only one of them is included in the final mRNA.

Retained Intron (RI): This intron is not spliced out and remains in the mature transcript.

### 2.2. Identification of Splicing Factors for SRF and Regulatory Networks

RNA-seq data from patients with heart failure and those without were used to identify alternative splicing events in SRF using rMATS. The UpSet plot (UpSetR, version 2.0) [19,20,21,22,23,24,25,26,27,28,29,30,31,32,33,34,35,36,37,38,39] was used to visualize intersecting sets among the five types of alternative splicing events. Moreover, splicing factors were retrieved for SRF from the RBPmap (https://rbpmap.technion.ac.il, accessed on 7 June 2025) using SRF transcript (NM_001292001.2: NM_003131.4) exon + flanking intron sequences [20,21].

**SRF exon + flanking intron sequences**

…… . . . . . upstream intron (200–500 bp) . . . . . .

[== EXON ==]

… . . . . . downstream intron (200–500 bp) . . . . . .

…… Dotted lines represent flanking intronic regions (e.g., ±200–500 bp of a specified length).

[====== EXON ======] represents the SRF exon sequence.

We conducted a correlation analysis using the Hmisc (version 5.2.3) R package 3.5.1, between splicing factors and SRF using the Pearson correlation method. Correlation coefficient > 0.7 and a *p*-value < 0.05 were considered statistically significant. These selected pairs were then imported into Cytoscape (version 3.6.0) to construct the regulatory interaction network. Among the correlated splicing factors, we identified the top six showing the strongest associations with SRF. Scatterplots were generated to visualize and evaluate the highest correlations between SRF and the respective splicing factors in heart failure vs. healthy individuals. In addition, the Hmisc (version 5.2.3) R package was used to find Spearman correlations between splicing factors and SRF (correlation coefficients > 0.4 and *p* ≤ 0.05). The expression profiling boxplot of selected splicing factors in samples from healthy controls and patients with heart failure was assessed. We also identified the top six splicing factors with the strongest positive correlations with SRF. Scatterplots were created for all factors to demonstrate expression patterns between heart failure and control samples. Boxplots were set up to illustrate the differential expressions of leading splicing factors, and statistical significance was assessed using Student’s *t*-test with *p* ≤ 0.05. Moreover, chromosomal localization of the correlated splicing factors was visualized by a Circos plot to assess genome-wide localization [20,21].

### 2.3. Functional Enrichment Analysis of Genes with Strong Correlations with SRF

ShinyGO 0.82 was used for KEGG, Gene Ontology, and enrichment analyses. The genes that were correlated with SRF were identified, and their presence was further screened using the R Script in the datasets PRJNA678360, PRJNA198165, and PRJNA477855 during splicing events. Subsequently, enrichment analysis was performed. The *p*-value 0.05 was used as a cutoff to define significant enrichment [22,23,24].

## 3. Results

### 3.1. RNA-Seq Data from the PRJNA198165 Dataset, Which Includes Four Patient Groups

**Figure 1:**
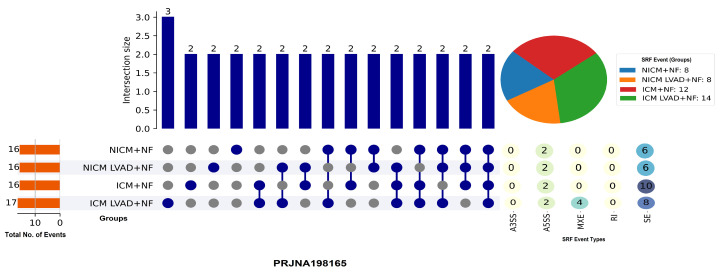
UpSet plot depicting splicing event among cardiomyopathy subgroups (NICM/ICM with or without LVAD). The SE events were the most common, with limited overlap among groups, suggesting distinct splicing patterns influenced by disease etiology and mechanical unloading.

NICM+NF, NICM LVAD+NF, ICM+NF, and ICM LVAD+NF were studied. Using an UpSet plot to visualize splicing event intersections, we identified that skipped exon (SE) events were the most prevalent form of alternative splicing across all groups, with a notable enrichment in certain intersections involving ICM samples. The total number of splicing events was slightly higher in the ICM LVAD+NF group (17 events), compared to the other groups (with 16 events each), implying that mechanical unloading via LVAD may enhance or reveal splicing variability, particularly in ischemic cardiomyopathy Figure 1. Intersection analysis revealed minimal overlap between groups, with most common events occurring in only two circumstances, highlighting the context-specific regulation of alternative splicing in heart failure. These findings highlight the importance of exon skipping as a major mechanism of transcriptomic remodeling in the failing human heart, implying that both disease subtype and LVADs contribute to splicing dynamics [25,26,27,28,29,30,31,32,33,34,35,36,37,38,39,40].

**Figure 2:**
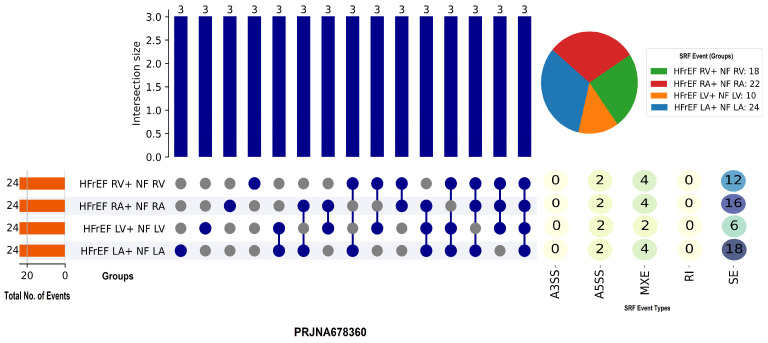
The UpSet plot depicts splicing event distribution across heart failure with reduced ejection fraction (HFrEF) subgroups, stratified by heart chamber (LA, LV, RA, RV). Skipped exon (SE) events were abundant across all groups, with a total of 18 events. The groups shared a consistent number of events (24 each), and intersection analysis revealed recurring triplet overlaps, suggesting shared splicing changes across heart chambers in HFrEF.

To investigate chamber-specific splicing patterns in heart failure with a reduced ejection fraction (HFrEF), we examined RNA-seq data from the PRJNA678360 dataset, which included samples from the left atrium (LA), left ventricle (LV), right atrium (RA), and right ventricle (RV). Each group had the same number of splicing events (*n* = 24), allowing for direct comparison across chambers. Skipped exon (SE) events were the most prominent type observed (n = 18), followed by mutually exclusive exons (MXE, n = 6). No events were observed in the A3SS, A5SS, or retained intron (RI) categories. Intersection analysis revealed consistent overlap between three events per intersection across multiple chamber combinations, indicating a subset of shared splicing events despite anatomical and functional differences Figure 2. These findings suggest that SE-driven splicing changes are a common characteristic of HFrEF across cardiac chambers, pointing to a potentially conserved splicing response in the failing human heart.

**Figure 3:**
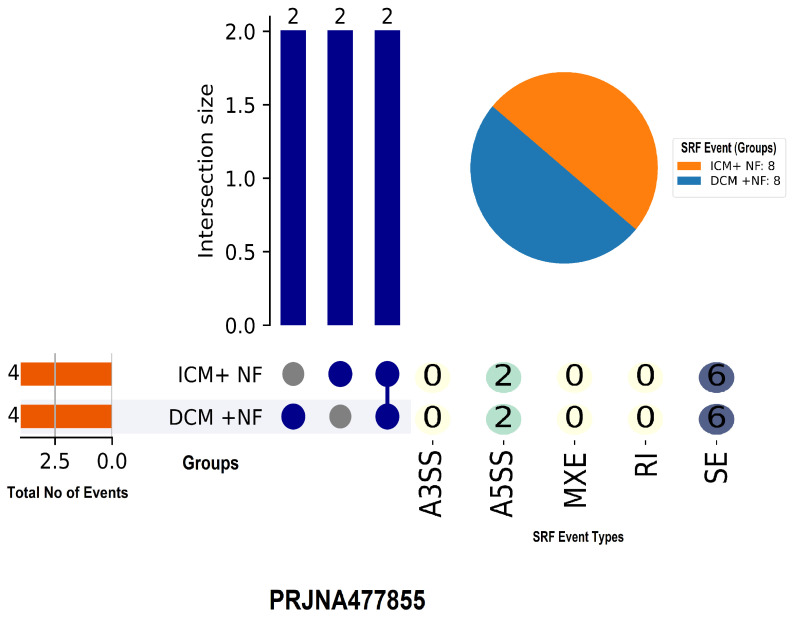
UpSet plot depicting alternative splicing events in dilated (DCM+NF) and ischemic (ICM+NF) cardiomyopathy from dataset PRJNA477855. The SE events were prominent, with complete overlapping between groups, suggesting shared splicing alterations in the absence of mechanical unloading.

We examined the splicing profiles of ischemic (ICM) and dilated (DCM) cardiomyopathy using RNA-seq data from the PRJNA477855 dataset, which contains the two groups ICM+NF and DCM+NF, each with eight samples. Each group had four identified splicing events. The most common splicing type was skipped exon (SE) events (n = 6), followed by alternative 5′ splice site (A5SS) events (n = 2). There were no occurrences discovered in the A3SS, MXE, or RI categories Figure 3.

Intersection analysis revealed that all events were shared by the two groups (intersection size = 2), indicating a high level of overlap in splicing patterns between ICM and DCM in the absence of mechanical unloading (reducing the heart’s workload using devices like LVADs). These data suggest that, while overall splicing activity is low in these situations, SE events may play an important role in transcriptome variations amongst cardiomyopathy subtypes.

We analyzed the splicing profiles of ICM and DCM using RNA-seq data from the PRJNA477855 dataset, which is divided into the two groups ICM+NF and DCM+NF, each with eight samples. Each group contained four identified splicing events. The most prevalent splicing type was SE events (n = 6), followed by A5SS events (n = 2). No occurrences were found in the A3SS, MXE, or RI categories.

### 3.2. An Overview of Alternative Splicing Events in Serum Response Factor

**Figure 4:**
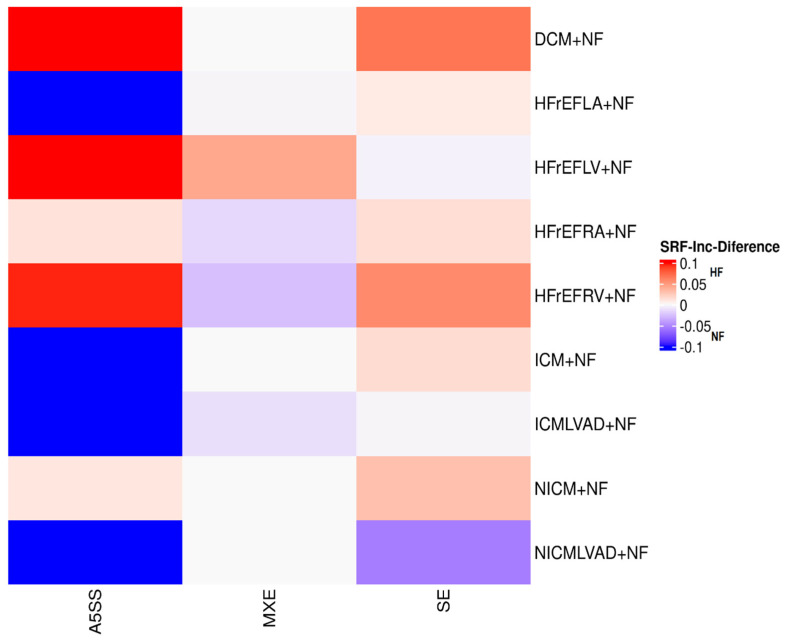
Differences in SRF-associated alternative splicing event inclusion between heart failure (HF) and non-failure (NF) patient groups across various cardiomyopathy subtypes. The heatmap shows differences in inclusion levels (Inc-Diff) for three main kinds of alternative splicing events: A5SS, MXE, and SE. The color red indicates higher inclusion in HF samples, while blue indicates higher inclusion in NF samples. Clustering and group-wise comparisons exhibit condition-specific splicing changes involving SRF, suggesting the regulatory role of SRF isoforms in HF pathogenesis.

To investigate the impact of alternative splicing regulation via SRF in heart failure, we compared the inclusion levels of SRF-associated splicing events between the HF and NF groups across multiple clinical subtypes. Figure 4 shows that splicing event types such as A5SS and SE displayed marked inclusion differences in specific patient groups. Notably, A5SS events were more prevalent in DCM+NF and HFrEF LV+NF groups, while reduced inclusion was observed in NICM+NF and NICMLVAD+NF groups. Similarly, SE events were predominantly higher in HF groups compared to NF groups.

These subtype-specific splicing signatures show that SRF may be regulated differently in heart failure, contributing to clinical heterogeneity. This validates our consensus clustering results and emphasizes the role of splicing factors in driving transcriptomic alterations in cardiomyopathy. The exonic coordinates of SRF during splicing are provided in Table 2.

### 3.3. Functional Enrichment Analyses

**Figure 5:**
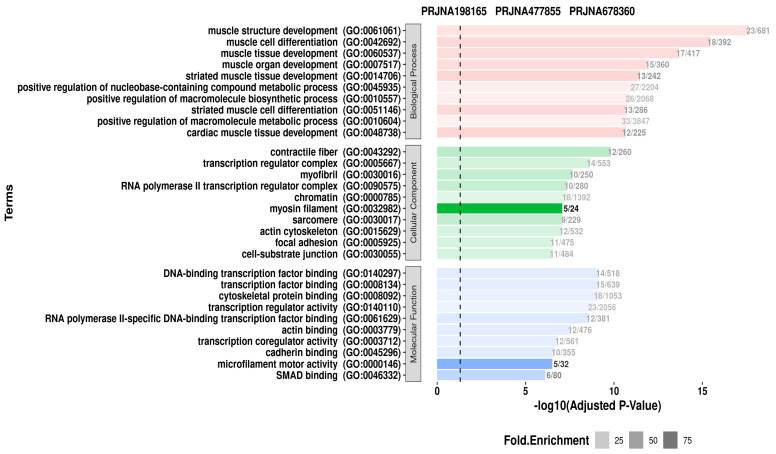
Gene Ontology (GO) enrichment analysis of genes correlated with SRF and detected in splicing datasets (PRJNA678360, PRJNA198165, PRJNA477855).

GO enrichment was performed using the cluster Profiler (v4.4.4) and org.Hs.eg.db (v3.15.0) R packages. The bar plot illustrates the Gene Ontology (GO) enrichment analysis of differentially expressed genes across transcriptomic datasets: PRJNA198165, PRJNA477855, and PRJNA678360. GO terms are categorized into three major categories: Biological Process, Cellular Component, and Molecular Function (Figure 5).

The *x*-axis indicates the significance of enrichment as −log10(adjusted *p*-value), and dot size within the bars indicates the fold enrichment values, providing a quantitative measure of over-representation. Highly enriched terms in the Biological Process category (pink bars) include muscle structure development, muscle cell differentiation, and muscle tissue development, demonstrating that muscle-related pathways are consistently engaged across datasets. These include processes such as the positive regulation of metabolic and biosynthetic processes, which further support the activation of anabolic and differentiation pathways relevant to muscle biology and tissue remodeling.

Muscle structure development, muscle cell differentiation, and muscle tissue development are among the most highly enriched terms in the Biological Process category (pink bars), indicating that muscle-related pathways are consistently active across datasets. The positive regulation of metabolic and biosynthetic processes promotes the activation of anabolic and differentiation pathways involved in muscle biology and tissue remodeling.

In the Cellular Component category (green bars), enriched GO terms such as myosin filament, contractile fiber, myofibril, and sarcomere emphasize the presence and activity of structural components required for muscle contraction. The dataset PRJNA477855 shows particularly high enrichment in the myosin filament (5 genes out of 24), indicating its important role in the contractile mechanism.

The Molecular Function category (blue bars) focuses on the genes involved in DNA-binding transcription factor activity, actin binding, and microfilament motor activity. These functions represent regulatory and mechanical characteristics that are critical for cellular structure and gene expression control in muscle-related conditions. Overall, the enrichment profile of the datasets indicates the stringent transcriptional regulation of muscle development, cytoskeletal architecture, and signaling pathways, with dataset-specific variations improving our understanding of gene function in muscle physiology and related conditions. Supplementary Table S1 shows information about genes that have a strong connection with SRF.

### 3.4. KEGG Pathway Enrichment Analysis

**Figure 6:**
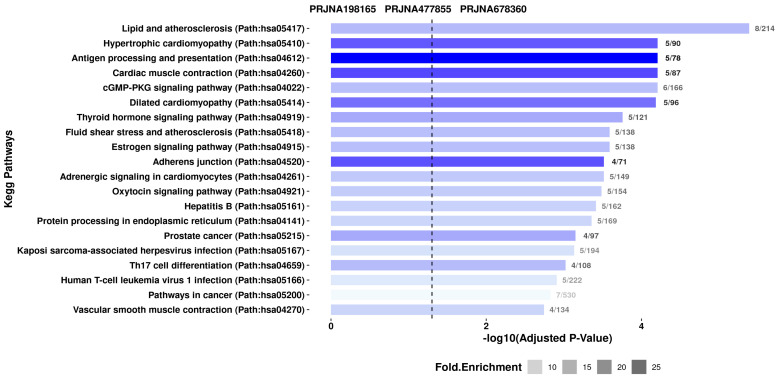
KEGG pathway enrichment analysis of the gene set showing significantly enriched biological pathways based on adjusted *p*-values. The *x*-axis represents the statistical significance as –log10 (adjusted *p*-value), while the color intensity of the bars reflects fold enrichment. Pathways related to cardiovascular function, immune response, hormone signaling, and cellular stress are prominently enriched, with the most significant being “lipid and atherosclerosis” and “antigen processing and presentation.” The numbers at the end of each bar indicate the ratio of input genes mapped to each pathway.

Figure 6 shows a bar plot of KEGG pathway enrichment results for genes that are differentially expressed in three transcriptome datasets. The *x*-axis indicates the −log10 (adjusted *p*-value), showing the statistical significance of pathway enrichment, and the dot size reflects fold enrichment. Larger dots imply greater enrichment. The *y*-axis lists KEGG pathway names, with bars colored according to their relevance across datasets.

There are several highly enriched pathways related to cardiovascular disease. They are lipid and atherosclerosis (hsa05417), hypertrophic cardiomyopathy (hsa05410), cardiac muscle contraction (hsa04260), dilated cardiomyopathy (hsa05414), and vascular smooth muscle contraction (hsa04270); these pathways have gene sets that have essential functions in processes of heart disease. Lipid and atherosclerosis comprise the most enriched pathway (8 out of 214 genes), with a crucial function in vascular remodeling and inflammation.

The immune and signaling process is also well represented. The antigen processing and presentation (hsa04612) pathway is highly enriched (5/78 genes), particularly in dataset PRJNA477855, indicating its potential role in immune regulation. Other highly augmented signaling pathways are estrogenic signaling, cGMP-PKG, adrenergic signaling in cardiomyocytes, and thyroid hormone signaling, indicating the regulation of gene expression by hormonal and second messenger pathways.

In addition, the enrichment profile also contains cancer-related pathways such as Kaposi sarcoma-associated herpesvirus infection (hsa05167), prostate cancer (hsa05215), and cancer pathways (hsa05200), indicating potential oncogenic overlap or common molecular processes. Overall, we report from this analysis that the transcriptomic patterns from the three datasets are generally shown to be highly correlated with cardiovascular function, immune activity, and oncogenic signaling, providing valuable insight into the Biological Processes underlying cardiovascular disease therapeutic targets.

### 3.5. Landscape of Splicing Factors in Heart Failure

**Figure 7:**
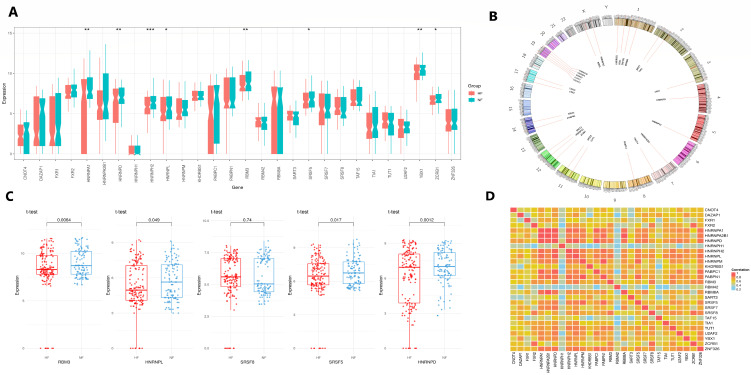
Differential expression and genomic organization of splicing factors. (**A**) A boxplot showing the expression levels of key splicing factors in heart failure vs. non-heart failure groups/ Significant differences are marked (* *p* < 0.05, ** *p* < 0.01, *** *p* < 0.001). (**B**) Circos plot showing chromosomal locations of differentially expressed splicing factors across the genome. (**C**) Boxplots showing five significantly dysregulated splicing factors (*FUS*, *HNRNPA1*, *SRSF1*, *SRSF3*, *HNRNPD*), with their respective *p*-values. (**D**) A heatmap showing pair-wise correlations among the splicing factors, with co-expressed gene clusters (red = positive correlation; blue = negative correlation; yellow = low correlation).

We examined the expression profiles of known splicing factors in two groups (heart failure vs. non-heart failure). As depicted in Figure 7A, multiple splicing-related genes displayed significant differences in expression between the groups, indicating a global alteration in the splicing mechanism. Genes such as *FUS*, *HNRNPA1*, *SRSF1*, *SRSF3*, and *HNRNPD* were particularly notable for their differential expression (*p* < 0.05), suggesting their potential involvement in aberrant splicing regulation.

The chromosomal locations of these differentially expressed splicing factors are illustrated in the Circos plot in Figure 7B, where they are evenly distributed in most chromosomes, showing their broad distribution. This genomic dispersion indicates that the observed splicing dysregulation is not confined to a specific chromosomal locus but reflects a systemic transcriptional shift.

We further validate the expression changes in five highly significant splicing factors through detailed boxplots in Figure 7C. These genes demonstrated consistent and statistically robust alterations between the groups, reinforcing their role in the altered splicing landscape.

Correlation analysis among the splicing factors, shown in Figure 7D, revealed distinct clusters of co-expressed genes. Strong positive correlations were observed among several heterogeneous nuclear ribonucleoproteins (HNRNPs) and serine/arginine-rich splicing factors (SRSFs), suggesting their potential co-regulation or participation in common splicing complexes or pathways.

### 3.6. Regulatory Network of Splicing Factors and Their Correlation with SRF

**Figure 8:**
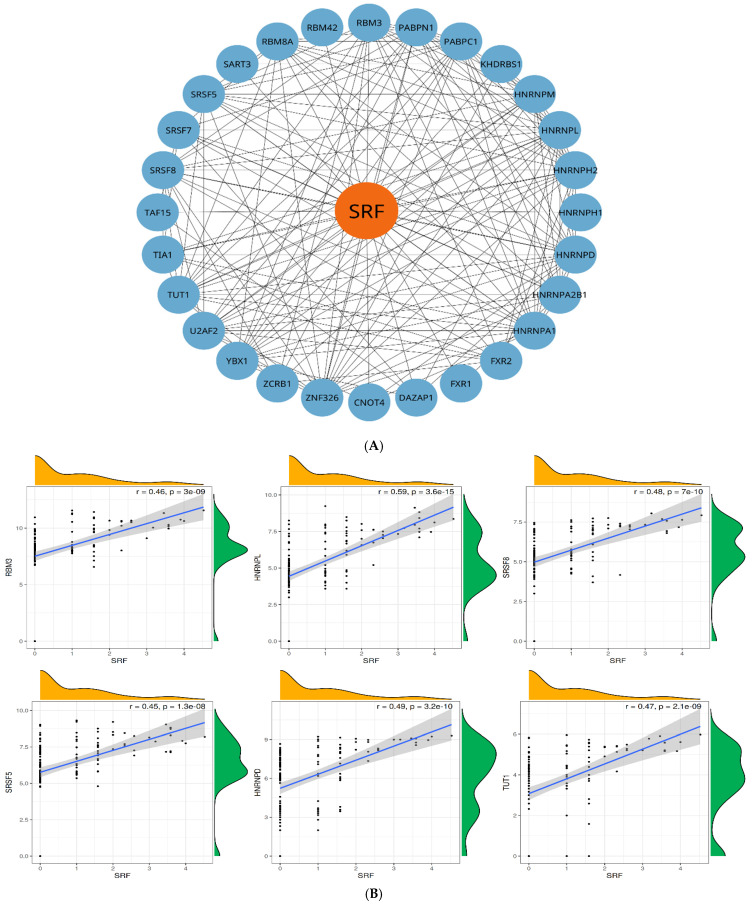
(**A**) SRF interactome network with splicing factors/RNA-binding proteins (RBPs). Network plot depicting the serum response factor (SRF, orange) interacting with many RNA-binding proteins (blue nodes). The extreme connectivity density is a sign of the SRF’s regulatory domination in RNA processing pathways. (**B**) Correlation analysis between SRF expression and RNA-binding/splicing factors. Scatter plots show the relationship between SRF and six genes: RBM3, HNRNPL, SRSF8, SRSF5, HNRNPD, and TUT1.

To identify key regulatory interactions between splicing factors, we constructed an SRF-focused splicing factor interaction network. As illustrated in Figure 8A, SRF interacts with a dense network of known splicing factors, indicating its potential integrative role in RNA splicing regulation. The network comprises several splicing factors, including HNRNPs (HNRNPA1, HNRNPM, HNRNPD, HNRNPH1, HNRNPH2, HNRNPL), serine/arginine-rich splicing factors (SRSFs) (SRSF5, SRSF7, SRSF8), and other essential splicing regulators such as U2AF2, PABPN1, TIA1, YBX1, KHDRBS1, and DAZAP1. The high connectivity among these factors suggests a highly integrated splicing regulatory network, where SRF can serve as a central node to connect disparate spliceosomal components.

This interaction map underscores the complex regulatory crosstalk among splicing factors and supports the hypothesis that splicing regulation is orchestrated through interconnected modules rather than isolated pathways.

All six splicing factors showed a substantial positive correlation with SRF expression (*p* < 0.001), suggesting potential co-regulation or transcriptional association. The strength of correlation varied across genes. HNRNPL formed a moderately strong correlation with SRF (r = 0.59, *p* = 3.6 × 10^−15^), followed by HNRNPD (r = 0.49) and SRSF8 (r = 0.48). RBM3, SRSF5, and TUT1 also showed moderately strong correlations with R-values ranging from 0.45 to 0.47. These findings suggest that increased SRF expression is associated with the elevated expression of multiple splicing regulators/RNA-binding proteins (Figure 8B).

The correlations observed might either indicate a role of SRF in the modulation of post-transcriptional processes or might imply that SRF functions in a wider gene regulatory network that affects RNA splicing or stability. These interactions need to be examined further, especially in the context of cellular differentiation, stress responses, or disease states such as cancer or neurodegeneration, where both SRF and RNA processing pathways play critical roles. Overall, this analysis provides evidence of SRF’s involvement in regulating genes essential for RNA metabolism.

The correlations observed could be indicative of SRF’s participation in modulating post-transcriptional processes or could imply that SRF functions in a larger gene regulatory network affecting RNA stability or splicing. These interactions need further exploration, especially in the context of cellular differentiation, stress response, or disease states such as cancer or neurodegenerative disease, where SRF as well as RNA processing pathways are of central importance. This overall analysis corroborates SRF involvement in gene regulation that is essential for RNA metabolism.

## 4. Discussion

Alternative splicing is an important process that regulates gene expression and increases proteome diversity, particularly in organs with high functional complexity, such as the heart [3]. We present the first comprehensive examination of SRF-related alternative splicing events in human failing hearts, utilizing publicly available RNA-seq datasets. Our study examined a variety of cardiac disease conditions, including ICM, NICM, DCM, and HFrEF, as well as samples with and without left ventricular assist devices.

Our findings revealed that SRF undergoes substantial alternative splicing, with different patterns depending on disease subtype and anatomical region. Skipped exon (SE) events were consistently the most common across all datasets, followed by mutually exclusive exons (MXEs) and alternative 5′ splice site (A5SS) events. These findings are consistent with previous research, which found that SE is the most common alternative splicing process in mammalian tissues [13,25,26].

Importantly, some SRF exons contributed to this splicing process under multiple circumstances, implying functional significance. The most common altered exons were Exon 2 (chr6: 43,173,847–43,174,113), Exon 4 (chr6: 43,176,548–43,176,667), and Exon 5 (chr6: 43,178,294–43,178,485). These exons are located near or within the range of the MADS-box domain, which is the core DNA-binding region of SRF that is important for recognizing CArG boxes in target gene promoters. Skipping these exons may result in a loss or change in DNA-binding capacity, which may affect the ability of SRF to regulate cardiac-specific genes, for example, MYH6, ACTA1, and ANKRD1. Moreover, dysregulation of these exons might result in shorter, faulty, or dysfunctional SRF isoforms that are accountable for pathological remodeling [27,28,29,30].

Disease-specific splicing patterns were most visible in the ICM and HFrEF groups, where SRF SE and A5SS events were most prominent. For example, the ICM+LVAD group displayed 17 unique SRF-associated splicing events, which is slightly higher than the ICM or NICM groups without LVAD support. This indicates that mechanical unloading with LVAD may not only relieve hemodynamic stress but also impact the transcriptome by unmasking or modulating splicing events [31]. Similarly, in the chamber-specific analyses of the HFrEF cohort, consistent SE events were observed across left atrium (LA), right atrium (RA), left ventricle (LV), and right ventricle (RV) tissues, highlighting conserved splicing changes throughout the diseased heart [28,29,30,31,32].

This study also explored splicing factors potentially regulating SRF expression or activity. Using RBPmap and correlation analysis, we discovered robust associations between SRF and several well-characterized splicing regulators. Notably, HNRNPL exhibited the strongest moderate correlation (r = 0.59, *p* = 3.6 × 10^−15^), followed by HNRNPD, SRSF8, SRSF5, RBM3, and TUT1. These splicing factors are involved in exon inclusion/exclusion decisions, splice site recognition, and transcript stability.

The observed co-expression patterns suggest that SRF may be a component of a feedback regulatory network with these splicing factors [33,34,35,36,37,38,39,40,41,42]. For example, HNRNPL and HNRNPD are part of the heterogeneous nuclear ribonucleoprotein (hnRNP) family, which antagonizes exon entry in several situations. Their high correlation with SRF implies the possibility that they are either downstream targets of SRF-mediated transcription or control the splicing of SRF itself. Meanwhile, SRSF5 and SRSF8 are serine/arginine-rich splicing factors that encourage the inclusion of exons, indicating a potentially balanced regulatory model in which both enhancer and silencer splicing elements are at play [33,34,35,36].

The interaction network analysis also showed that SRF is at the center of a dense regulatory network that includes several RNA-binding proteins (RBPs), supporting the role of SRF in regulating post-transcriptional processes in the heart. The Circos plot and splicing factor expression heatmap revealed that these genes were widely but differently expressed in heart failure versus non-failing tissues (Figure 7A–D). These patterns highlight a system-wide shift in the splicing process, possibly driven by inflammation, stress, or neurohormonal activation in failing hearts.

Functionally, SRF-associated genes involved in splicing events were enriched for muscle structure development, actin binding, sarcomere organization, and contractile fiber formation, as determined by Gene Ontology (GO) enrichment analysis. The similarity of these terms between datasets highlights the precedence of alternative splicing in maintaining or disrupting cardiac contractility and structure. In the analysis of the KEGG pathway, the enrichment of hypertrophic cardiomyopathy (HCM), dilated cardiomyopathy (DCM), vascular smooth muscle contraction, and lipid metabolism was observed. More importantly, immune-related pathways such as antigen processing and presentation were also enriched, indicating an intersection between immune signaling and splicing regulation in the failing myocardium [1,2,37,38].

From the translational and clinical perspective, this research has multiple implications. First, the observation of consistent, disease-specific SRF exon splicing patterns suggests that such isoforms could be utilized as biomarkers to separate heart failure subtypes or to monitor response to treatment with LVADs. Second, targeting splicing factors that regulate SRF may offer new therapeutic opportunities. For example, antisense oligonucleotides (ASOs) or small molecules that modulate exon inclusion/exclusion have shown promise in other diseases like spinal muscular atrophy and could be repurposed for heart failure if important splicing events are validated [38,39,40,41,42].

Nevertheless, there are a few caveats in this research. The use of bulk RNA-seq data limits our resolution, because we could not determine the cell type(s) where the SRF splicing events occurred—whether cardiomyocytes, fibroblasts, endothelial cells, or infiltrating immune cells were involved. Additionally, although rMATS is a powerful tool for splicing analysis, it may be incapable of detecting multistep events such as recursive splicing or those involving cryptic splice sites. Functional analyses, for example, CRISPR-induced exon deletion, isoform-specific knockdown, or RNA immunoprecipitation (RIP), must be used to validate the functional relevance of these findings.

## 5. Conclusions

In conclusion, this study reveals that SRF undergoes significant alternative splicing in cardiac disease failure, particularly in exons critical to its DNA-binding function. These splicing patterns vary by disease subtype and cardiac chamber and are strongly associated with specific splicing regulators. The functional enrichment of correlated genes supports the role of these SRF splicing events in cardiac development, structure, and function. Together, our findings position SRF not just as a transcriptional regulator, but also as a key integrated nodal point in the splicing-transcription regulatory network of the human heart. Future research should explore the therapeutic potential of modulating these splicing events to mitigate cardiac pathologies associated with the progression of heart failure. Specifically, in Heart Failure with Preserved Ejection Fraction (HFpEF) which is prevalent with advance age [43].

## Figures and Tables

**Table 1:** The details of cardiovascular disease used in the analysis of skipped exons.

Sequence Read Archive ID	Types of Cardiovascular Disease
PRJNA198165	Ischemic Cardiomyopathy (ICM), Non-ischemic cardiomyopathy (NICM), ICM+ Left ventricular assist device (LVAD), and NICM+LVAD vs. Non-failing group
PRJNA678360	Heart failure with reduced ejection fraction (HFrEF) vs. Non-failing group
PRJNA477855	Dilated cardiomyopathy and Ischemic cardiomyopathy vs. Non-failing group

**Table 2:** The exonic coordinates of SRF during splicing.

Condition	Exon Coordinates (chr6, +Strand)	Types of ASE	SRA ID
SRF NF and SRF ICM	43,173,847–43,174,113 (Exon 2), 43,178,806–43,178,882 (Exon 6), 43,176,548–43,176,667 (Exon4), 43,178,294–43,178,485 (Exon 5), 43,175,706–43,175,967 (Exon3)	SE	PRJNA198165
SRF NF and ICMLVAD	43,172,396–43,172,451 (Exon 1), 43,172,396–43,172,578 (Exon 1), 43,173,847–43,174,113 (Exon 2)	A5SS	
	43,173,847–43,174,113 (Exon 2), 43,175,706–43,175,967 (Exon 2), 43,176,548–43,176,667 (Exon 4), 43,178,294–4,317,848 (Exon 5)	MXE	
	43,173,847–43,174,113 (Exon 2), 43,175,706–43,175,967 (Exon 3), 43,176,548–43,176,667 (Exon 4), 43,173,847–43,174,113 (Exon 2), 43,176,548–43,176,667 (Exon 4), 43,178,806–43,178,882 (Exon 6), 43,175,706–43,175,967 (Exon 3), 43,176,548–43,176,667 (Exon 4), 43,178,294–43,178,485 (Exon 5), 43,176,548–43,176,667 (Exon 4), 43,178,294–43,178,485 (Exon 5), 43,178,806–43,178,882 (Exon 6)	SE	
SRF NF and NICM	43,172,396–43,172,451 (Exon 1), 43,172,396–43,172,578 (Exon 7), 43,173,847–43,174,113 (Exon 2)	A5SS	
SRF NF and NICMLVAD	43,173,847–43,174,113 (Exon 2), 43,175,706–43,175,967 (Exon 2), 43,176,548–43,176,667 (Exon 4), 43,178,806–43,178,882 (Exon 6), 43,178,294–43,178,485 (Exon 5), 43,178,806–43,178,882 (Exon 6)	SE	
SRF NF and DCM	43,172,396–43,172,451(Exon 1), 43,172,396–43,172,578 (Exon 2), 43,173,847–43,174,113 (Exon 2)	A5SS	PRJNA477855
SRF NF and ICM	43,172,396–43,172,451(Exon 1), 43,172,396–43,172,578 (Exon 2) 2), 43,173,847–43,174,113 (Exon 2)	A5SS	
SRF NF and HFrEF LA	43,172,396–43,172,451(Exon 1), 43,172,396–43,172,578, 43,173,847–43,174,113 (Exon 2)	A5SS	PRJNA678360
SRF NF and HFrEF LA	43,173,847–43,174,113 (Exon 2), 43,175,706–43,175,967, 43,178,294–43,178,485 (Exon 5), 43,176,548–43,176,667 (Exon 4), 43,178,806–43,178,882 (Exon 6)	MXE	
SRF NF and HFrEF LA	43,173,847–43,174,113 (Exon 2), 43,175,706–43,175,967 (Exon 3), 43,176,548–43,176,667 (Exon 4), 43,178,294–43,178,485 (Exon 5), 43,178,806–43,178,882 (Exon 6), 43,176,548–43,176,667(Exon 4), 43,179,095–43,181,506 (Exon 7)	SE	
SRF NF and HFrEF RA	43,172,395–43,172,578, 43,172,395–43,172,451(Exon 1), 43,173,846–43,174,113 (Exon 2)	A5SS	
SRF NF and HFrEF RA	43,173,846–43,174,113 (Exon 2), 43,175,705–43,175,967 (Exon 3), 43,176,547–43,176,667 (Exon 4), 43,178,293–43,178,485 (Exon 5), 43,178,805–43,178,882 (Exon 6)	MXE	
SRF NF and HFrEF LV	43,172,396–43,172,451 (Exon 1), 43,172,396–43,172,578 (Exon 2), 43,173,847–43,174,113 (Exon 2)	A5SS	
SRF NF and HFrEF LV	43,173,847–43,174,113 (Exon 2), 43,175,706–43,175,967 (Exon 3), 43,176,548–43,176,667 (Exon 4), 43,178,294–43,178,485 (Exon 5)	MXE	
SRF NF and HFrEF LV	43,175,705–43,175,967 (Exon 3),43,176,547–43,176,667 (Exon 4)	SE	
SRF NF and HFrEF RV	43,172,395–43,172,578 (Exon 1), 43,172,395–43,172,451 (Exon 1), 43,173,847–43,174,113 (Exon 2)	A5SS	
SRF NF and HFrEF RV	43,175,705–43,175,967 (Exon 3), 43,176,547–43,176,667 (Exon 4), 43,178,293–43,178,485 (Exon 5), 43,178,805–43,178,882 (Exon 6)	MXE	
SRF NF and HFrEF RV	43,175,705–43,175,967 (Exon 3), 43,176,547–43,176,667 (Exon 4), 43,178,293–43,178,485 (Exon 5)	SE	

## Data Availability

The authors confirm that all data supporting the findings of this study are available within the article.

## Supplementary Materials

The following supporting information can be downloaded at https://www.mdpi.com/article/10.3390/genes16080947/s1. Supplementary Table S1 The details of genes which has strong correlation with SRF.
