# Supplementary material for: Alternative Splicing of Serum Response Factor Reveals Isoform-Specific Remodeling in Cardiac Diseases
Source: Genes (Basel). 2025 Aug 11;16(8):947. doi: 10.3390/genes16080947 (PMC12385735; doi:10.3390/genes16080947)
Supplement: Supplementary file 1 [file genes-16-00947-s001.zip › Supplementary Table 1.pdf]

Supplementary Table 1. The details of genes which has strong correlation with SRF

Gene	Full Name	Functional Role	SRF Correlation
FOS	FBJ Murine Osteosarcoma Viral Oncogene	Immediate-early response gene; regulates proliferation, apoptosis	Early SRF target gene via MAPK pathway
JUN	Jun Proto-Oncogene	Forms AP-1 complex; involved in proliferation, stress response	Immediate-early SRF target; ternary complex pathway
EGR1	Early Growth Response 1	Zinc-finger TF; regulates cell growth, development	Rapidly induced by SRF signaling
MYH9	Myosin Heavy Chain 9 (non-muscle)	Cytoskeletal motor protein; cellular tension regulation	Indirect, related to cytoskeletal feedback on SRF
MYH11	Myosin Heavy Chain 11 (smooth muscle)	Contractile protein in smooth muscle	SRF target gene; marker of smooth muscle differentiation
ACTA1	Actin Alpha 1 (skeletal muscle)	Major actin isoform in skeletal muscle	Actin gene, SRF-regulated
ACTA2	Actin Alpha 2 (smooth muscle)	Key smooth muscle contractile protein	Canonical SRF target gene
VCL	Vinculin	Focal adhesion protein; connects actin cytoskeleton to membrane	Cytoskeletal protein affecting MRTF/SRF signaling
CNN1	Calponin 1	Smooth muscle differentiation marker	SRF target, regulated via myocardin/MRTFs
TAGLN	Transgelin (SM22α)	Actin-binding protein in smooth muscle cells	Direct SRF target gene
GAP43	Growth Associated Protein 43	Involved in neural development and regeneration	Not a direct SRF target; minimal correlation
MRTFA	Myocardin-Related TF A	SRF co-factor, regulates smooth muscle and actin genes	Direct SRF co-activator
MRTFB	Myocardin-Related TF B	Same as MRTFA; acts redundantly in smooth/cardiac tissue	Direct SRF co-activator
ACTC1	Actin Alpha Cardiac Muscle 1	Cardiac muscle actin	Strong SRF target in cardiac tissue
MYH6	Myosin Heavy Chain 6 (alpha, cardiac)	Cardiac contractile protein	Transcription influenced by SRF and co-factors

MYH7	Myosin Heavy Chain 7 (beta, cardiac)	Cardiac muscle; slower contraction isoform	Indirect SRF association
TNNT2	Troponin T2 (cardiac)	Cardiac-specific sarcomeric protein	Regulated during cardiac development; possible indirect SRF target
TPM1	Tropomyosin 1	Actin filament binding protein, regulates contraction	Smooth muscle and cardiac expression regulated by SRF
CNN3	Calponin 3	Cytoskeletal protein, neural and smooth muscle roles	Less characterized, potential SRF pathway target
NPPA	Natriuretic Peptide A	Regulates blood pressure and volume	Indirect regulation through cardiac SRF activity
NPPB	Natriuretic Peptide B	Cardiac hormone, stress-responsive	Indirectly regulated; responsive to mechanical stress via SRF
CYR61	Cysteine-Rich Angiogenic Inducer 61	ECM signaling and angiogenesis	Target of MRTF-SRF under stress
FHL1	Four and a Half LIM Domains 1	Muscle development and cytoskeleton organization	Interacts with SRF in cardiac/skeletal muscle
FHL2	Four and a Half LIM Domains 2	Signal transducer in muscle tissues	Enhancer of SRF transcriptional activity
MYOCD	Myocardin	Master regulator of smooth and cardiac muscle genes	Major SRF co-activator
MKL1	Megakaryoblastic Leukemia 1	Same as MRTFA	SRF co-factor
MKL2	Megakaryoblastic Leukemia 2	Same as MRTFB	SRF co-factor
RhoA	Ras Homolog Family Member A	Regulates actin polymerization	Upstream regulator of MRTF/SRF pathway
GATA4	GATA Binding Protein 4	Cardiac transcription factor	Cooperates with SRF in heart gene expression
NKX2-5	NK2 Homeobox 5	Essential for cardiac development	Synergizes with SRF in heart morphogenesis
TCF12	Transcription Factor 12	Regulates differentiation, dimerizes with other bHLH factors	Minimal direct SRF correlation
PI3K/AKT pathway	Phosphoinositide 3-Kinase / Protein Kinase B Pathway	Regulates survival, metabolism	Modulates SRF activation via upstream signaling
MAPK pathway	Mitogen-Activated Protein Kinase Pathway	Stress, proliferation, differentiation signaling	Activates TCF-SRF complex via ELK1

YAP/TAZ	Yes-Associated Protein / WWTR1	Hippo pathway effectors, mechanotransduction	Interact with MRTFs and influence SRF outputs
ELK1	ETS Like-1	TCF that partners with SRF	Classical ternary complex component with SRF
ELK4	ETS Like-4	SAP-1, partner in SRF complex	Ternary complex with SRF, MAPK-responsive
FOxG1	Forkhead Box G1	Brain development TF	No known direct correlation with SRF
NFATC3	Nuclear Factor of Activated T Cells 3	T cell activation, hypertrophy regulator	Indirect crosstalk with SRF
NFATC4	Nuclear Factor of Activated T Cells 4	Similar to NFATC3	Indirect crosstalk with SRF
SRFBP1	SRF Binding Protein 1	Modulates SRF activity	Binds SRF, may modulate transcription
SMYD1	SET and MYND Domain Containing 1	Muscle differentiation histone methyltransferase	Co-regulator with SRF in muscle gene expression
TRIM55	Tripartite Motif Containing 55 (MURF2)	Muscle-specific E3 ubiquitin ligase	May affect SRF-regulated cytoskeleton turnover
TRIM24	Tripartite Motif Containing 24	Chromatin regulator	Unknown correlation with SRF
TRIM32	Tripartite Motif Containing 32	E3 ubiquitin ligase in muscle	Indirect effect on SRF pathway
ASCC3	Activating Signal Cointegrator Complex Subunit 3	Transcription coactivator complex	No direct SRF role established
ATF6	Activating Transcription Factor 6	ER stress response	Minimal or indirect SRF crosstalk
CEBPB	CCAAT Enhancer Binding Protein Beta	Inflammatory and stress gene regulator	Minimal SRF interaction
CREBBP	CREB Binding Protein	Histone acetyltransferase, transcriptional coactivator	General coactivator; may influence SRF indirectly
GTF2F1	General Transcription Factor IIF Subunit 1	RNA Pol II transcription factor	Core transcriptional machinery, indirect SRF relevance
GTF2I	General Transcription Factor II-I	Multifunctional TF; binds SRF motif	TFII-I known to interact with SRF-bound DNA

NFYA	Nuclear Transcription Factor Y Subunit Alpha	Histone-like DNA binding protein	Possible cooperation with SRF in some contexts
NCOR2	Nuclear Receptor Corepressor 2	Transcriptional repressor	No direct SRF link
PML	Promyelocytic Leukemia Protein	Nuclear body organizer, tumor suppressor	Indirect via transcriptional modulation
SRC	SRC Proto-Oncogene	Tyrosine kinase, integrator of adhesion signaling	Upstream regulator of cytoskeleton → SRF pathway
TEAD1	TEA Domain Transcription Factor 1	Partner of YAP/TAZ	Indirect SRF modulation via cytoskeletal dynamics
HSP90AA1	Heat Shock Protein 90 Alpha	Protein chaperone	May stabilize actin regulators affecting SRF
HSP90AB1	Heat Shock Protein 90 Beta	Constitutive HSP90 isoform	As above
HSPA1A/B	Heat Shock Protein Family A (Hsp70) Member 1A/1B	Chaperone, stress response	Indirect role in cell survival and SRF regulation
HSPA5	Heat Shock Protein Family A (Hsp70) Member 5	ER chaperone (BiP)	Minimal direct correlation
HSPA8	Heat Shock Protein Family A Member 8	Constitutive Hsp70	Indirect involvement via stress pathways
GAPDH	Glyceraldehyde-3-Phosphate Dehydrogenase	Glycolysis enzyme	Housekeeping gene, not SRF-regulated
PRDX1	Peroxiredoxin 1	Antioxidant enzyme	No established SRF connection
PELP1	Proline, Glutamate and Leucine Rich Protein 1	Nuclear receptor coactivator	Unknown SRF relevance
CTNNB1	Catenin Beta 1 (β-Catenin)	Wnt signaling, cell adhesion	May modulate SRF via mechanotransduction
TFII-I	Transcription Factor II-I (GTF2I)	Multifunctional TF	Known interaction with SRF at CArG boxes
FOXG1	Forkhead Box G1	Brain-specific TF	Minimal SRF relation
Gene	Full Name	Functional Role	SRF Correlation
FOS	FBJ Murine Osteosarcoma Viral Oncogene	Immediate-early response gene; regulates	Early SRF target gene via MAPK pathway

		proliferation, apoptosis	
JUN	Jun Proto-Oncogene	Forms AP-1 complex; involved in proliferation, stress response	Immediate-early SRF target; ternary complex pathway
EGR1	Early Growth Response 1	Zinc-finger TF; regulates cell growth, development	Rapidly induced by SRF signaling
MYH9	Myosin Heavy Chain 9 (non-muscle)	Cytoskeletal motor protein; cellular tension regulation	Indirect, related to cytoskeletal feedback on SRF
MYH11	Myosin Heavy Chain 11 (smooth muscle)	Contractile protein in smooth muscle	SRF target gene; marker of smooth muscle differentiation
ACTA1	Actin Alpha 1 (skeletal muscle)	Major actin isoform in skeletal muscle	Actin gene, SRF-regulated
ACTA2	Actin Alpha 2 (smooth muscle)	Key smooth muscle contractile protein	Canonical SRF target gene
VCL	Vinculin	Focal adhesion protein; connects actin cytoskeleton to membrane	Cytoskeletal protein affecting MRTF/SRF signaling
CNN1	Calponin 1	Smooth muscle differentiation marker	SRF target, regulated via myocardin/MRTFs
TAGLN	Transgelin (SM22α)	Actin-binding protein in smooth muscle cells	Direct SRF target gene

The list of gene found after screening in data set PRJNA678360, PRJNA198165, and PRJNA477855

SRC, CREBBP, NKX2-5, HSP90AA1, CTNNB1, HSP90AB1. ATF6, TCF12, TPM1, TNNT2, TRIM32, TRIM55, GTF2I, GATA4, NCOR2, GAPDH, MRTFA, GTF2F1, CNN1, PELP1, MYOCD,MRTFB,NFATC3,ACTC1,PML,NFYA,HSPA8,NPPB,ASCC3,ACTA1,MYH7, CNN3,FHL2,MYH6,ELK1,TRIM24,FHL1,SMYD1,ACTA2,VCL,TEAD1,MYH11,FOS,NF ATC4,PRDX1,HSPA5, MYH9
